# Brain-machine interface to control a prosthetic arm with monkey ECoGs during periodic movements

**DOI:** 10.3389/fnins.2014.00417

**Published:** 2014-12-12

**Authors:** Soichiro Morishita, Keita Sato, Hidenori Watanabe, Yukio Nishimura, Tadashi Isa, Ryu Kato, Tatsuhiro Nakamura, Hiroshi Yokoi

**Affiliations:** ^1^Brain Science Inspired Life Support Research Center, The University of Electro-CommunicationsChofu, Japan; ^2^Department of Mechanical Engineering and Intelligent Systems, The University of Electro-CommunicationsChofu, Japan; ^3^Division of Behavioral Development, Department of Developmental Physiology, National Institute for Physiological SciencesOkazaki, Japan; ^4^Department of Physiological Sciences, School of Life Science, The Graduate University for Advanced Studies (SOKENDAI)Hayama, Japan; ^5^PRESTO, Japan Science and Technology AgencyKawaguchi, Japan; ^6^Division of Systems Research, Department of Systems Design, Faculty of Engineering, The Yokohama National UniversityYokohama, Japan; ^7^Integrative Brain Imaging Center, National Center of Neurology and PsychiatryKodaira, Japan

**Keywords:** brain-machine interfaces, electrocorticography, electromyography, prosthetic arm, reaching task

## Abstract

Brain–machine interfaces (BMIs) are promising technologies for rehabilitation of upper limb functions in patients with severe paralysis. We previously developed a BMI prosthetic arm for a monkey implanted with electrocorticography (ECoG) electrodes, and trained it in a reaching task. The stability of the BMI prevented incorrect movements due to misclassification of ECoG patterns. As a trade-off for the stability, however, the latency (the time gap between the monkey's actual motion and the prosthetic arm movement) was about 200 ms. Therefore, in this study, we aimed to improve the response time of the BMI prosthetic arm. We focused on the generation of a trigger event by decoding muscle activity in order to predict integrated electromyograms (iEMGs) from the ECoGs. We verified the achievability of our method by conducting a performance test of the proposed method with actual achieved iEMGs instead of predicted iEMGs. Our results confirmed that the proposed method with predicted iEMGs eliminated the time delay. In addition, we found that motor intention is better reflected by muscle activity estimated from brain activity rather than actual muscle activity. Therefore, we propose that using predicted iEMGs to guide prosthetic arm movement results in minimal delay and excellent performance.

## Introduction

Brain-machine interfaces (BMIs), which are a type of man-machine interface that provides a direct connection between the brain and external devices, can be divided into 2 types: input-type and output-type. An input-type BMI is used for the recovery of central nervous system function with an external device (Yokoi et al., 2012), while an output-type BMI is used for the intuitive control of an external device instead of the limbs. For patients with severe paralysis, such as those with amyotrophic lateral sclerosis, output-type BMIs offer a promising technology for the rehabilitation of upper limb function (Lebedev and Nicolelis, 2009).

In an output-type BMI, brain activities are measured from the sensory motor area in the cerebral cortex; these signals can be detected invasively or noninvasively. Invasive approaches usually include the use of a multichannel needle-shaped sensor that is inserted into the cerebral cortex. Noninvasive approaches include the use of electroencephalography, functional near-infrared spectroscopy, or functional magnetic resonance imaging. Noninvasive approaches are ideal because they have no clinical risk; however, their spatial resolution and signal-to-noise ratio are not suitable for practical control. As a result, many studies continue to focus on invasive approaches. The initial studies on BMI focused on invasive signal detection of brain activity, and they achieved highly successful control of a prosthetic hand (Velliste et al., 2008) with good spatial resolution and signal-to-noise ratios. However, degeneration and necrosis limit the long-term use of these invasive signal detection methods (Szarowski et al., 2003; Biran et al., 2005). To overcome this problem, an electrocorticography (ECoG) electrode was developed. This is an invasive signal detection method involving the use of a surface electrode on the cerebral cortex under the dura matter. Importantly, it has long-term stability with low clinical risk. Moreover, it shows precise spatial resolution with a good signal-to-noise ratio. ECoGs have been used to develop output-type BMI systems for two-dimensional cursor control and motion prediction of the upper arm (Schalk et al., 2007; Pistohl et al., 2008; Uejima et al., 2009; Yanagisawa et al., 2009; Chao et al., 2010; Yanagisawa et al., 2011).

We also developed a prosthetic arm that is controlled by a BMI with ECoGs (Sato et al., 2012). The subject was a monkey (*Macaca fuscata*) implanted with ECoG electrodes and then trained in a reaching task. The reaching task was performed periodically. Therefore, decoding could be achieved by phase estimation of the periodic movements. A decoder was constructed by machine learning to map between the ECoGs and motion states, which corresponded to the phases of periodic movement. We then tested whether the response delay of the prosthetic arm was controlled by the proposed method. We found that the latency (the time that elapsed between the monkey's actual motion and the prosthetic arm movement) was about 200 ms. Considering the primary delay that the prosthetic arm has as a robotic arm, it is desirable that the trigger event generated precedes the monkey's actual motion by about 200 ms.

Since muscle activity precedes changes in motion, and motor intentions can be detected more quickly, one potential way of improving the response of the BMI prosthetic arm could lie in decoding muscle activity. In other words, as the musculoskeletal system is the best “device” for achieving the brain's motor intentions, using the musculoskeletal system may be advantageous in optimizing BMIs. In fact, myoelectric prosthetic hands are already commercially available (Naidu et al., 2008), while BMI prosthetic hands are not in practical use. Unfortunately, the body image that is presented by a BMI prosthetic arm to the brain differs considerably from that presented by a natural arm because the former cannot reflect motor intentions as faithfully as the musculoskeletal system. An electromyogram (EMG) prosthetic arm estimates motor intentions from the activities of a patient's residual muscles, and it typically accomplishes more sophisticated motions than a BMI prosthetic arm. However, the results of our latest study (Yokoi et al., in press), in which we compared the muscle and brain activities of monkeys, suggested that EMG prosthetic arms might not always be superior to BMI robotic arms in the estimation of the brain's motor intentions. Specifically, during periodic movements, predicted muscle activity from brain activity maintains the periodicity rather than actual muscle activity. Moreover, it is difficult to estimate motor intention directly from brain activities as mentioned above. Therefore, estimating motor intentions with predicted muscle activity from brain activity is likely a better method than directly estimating brain activity or actual muscle activity. Therefore, we devised a method of controlling a BMI prosthetic arm based on the above ideas, and sought to experimentally confirm the validity of this method.

## Materials and methods

### Abstract level of motor intention

Motor intentions are divided into different types depending on their abstract level. As an example, consider a reaching motion, such as that in self-feeding in monkeys. This motion consists of the following movement sequences: reaching forearm to an object, grasping the object, and returning forearm while grasping the object.

At first, various types of physical measures, such as the EMGs of each muscle, the grip force, angular velocities of the joints, three-dimensional wrist positions, and hand postures can be determined. These are motor intentions of the lower abstract level. Next, based on the interpretations of these physical values, the movement phase (e.g., waiting, reaching, grasping, or resting) can be considered as the motor intention of the higher abstract level. Of course, the monkey's intention in performing the reaching movement is one of the motor intentions of a higher abstract level. In this study, we considered the motor intentions of this abstract level as task-oriented motor intentions. According to the theory of localization of brain functions, information from different abstract levels is processed in different parts of the cerebral cortex. Following this, the planning, control, and execution of voluntary motions are processed in the motor cortex. Moreover, a preceding study confirmed the correlation between the modulation of neurons in the primary motor cortex and muscle activity (Morrow and Miller, 2002). In this paper, the abstract level of motion intention is discussed based on the brain and muscle activity that was measured in a monkey's motor cortex.

### Experimental subject

A monkey (*M. fuscata*) implanted with EMG and ECoG electrodes was used as the experimental subject. EMG and ECoG signals were recorded simultaneously with a Neural Data Acquisition System MAP system (Plexon Inc., Dallas, TX, USA). EMG signals were recorded as auxiliary analog inputs on an OmniPlex system. Signals were low-pass filtered (250-Hz cutoff), and the signals were recorded with a 500-Hz sampling rate. The target muscles that are related to the locomotion of the upper limb and hand and were used to measure EMGs are listed in Table 1.

**Table 1:** **Target muscles for measuring electromyograms**.

**Target muscle**	**Mainly moving joint**
PM (Pectoralis Major)	Shoulder
DP (Deltoid Posterior)	
TLoH (Triceps Long Head)	Elbow
TLaH (Triceps Lateral Head)	
BLH (Biceps Long Head)	
B (Brachioradialis)	
ECR (Extensor Carpi Radialis)	Hand
EDC (Extensor Digitorum Communis)	Index, little finger
FDP (Flexor Digitorum Profundus)	Finger
FCU (Flexor Carpi Ulnaris)	Hand
APL (Abductor Pollicis Longus)	Thumb/Hand
AP (Adductor Pollicis)	Thumb

**Figure 1:**
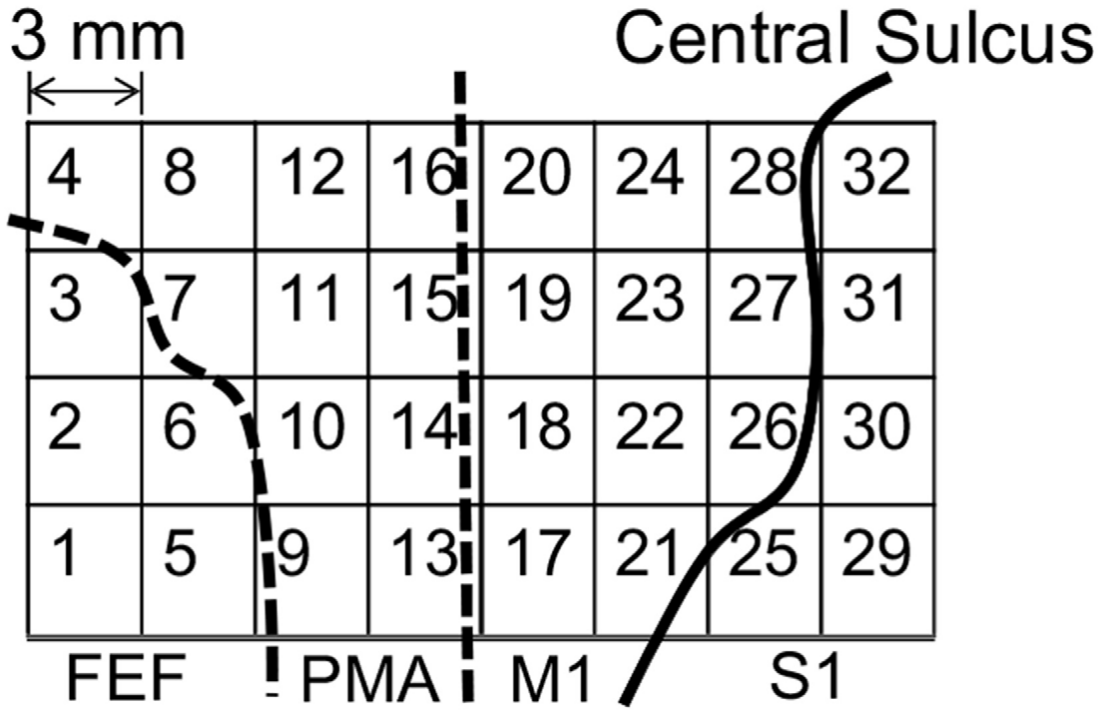
**Placement of the electrocorticogram (ECoG) electrodes around the motor cortex**. FEF, frontal eye field; PMA, premotor area; M1, primary motor cortex; S1, primary somatosensory cortex.

Figure 1 shows the placements of the ECoG electrodes. The target area was around the left motor cortex, including the frontal eye field, the premotor area, the primary motor cortex, and the primary somatosensory cortex.

### A prosthetic arm with an interference-based wire-driven mechanism

**Figure 2:**
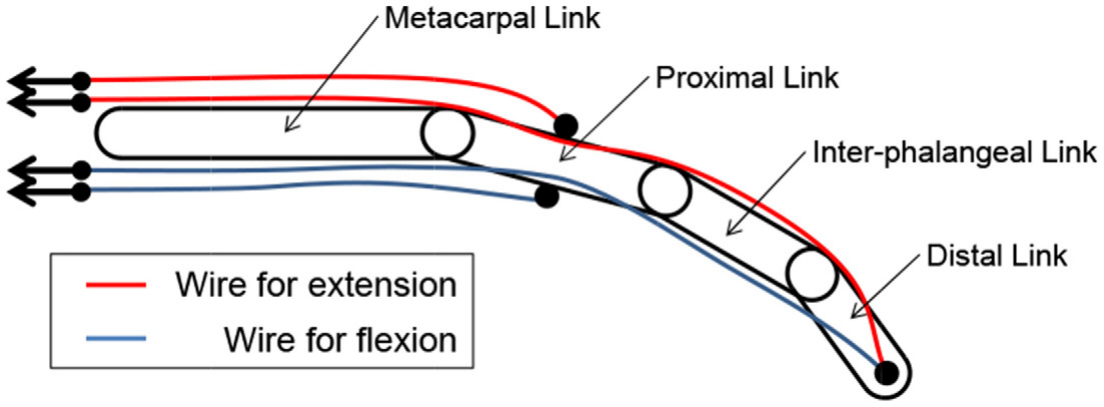
**Interference-based wire-driven mechanism of maniphalanx joints**.

An interference-based wire-driven mechanism was applied to the prosthetic arm to create a balance between the high grip force and high degree of motion retaining lightness. This mechanism involves use of wires to transmit driving force from the actuators. It considers the weight saving of the prosthetic hands attached to the patient's stump since it enables separation of the power sources and prosthetic hands. Figure 2 shows the interference-based wire-driven mechanism of the maniphalanx joints that are designed for the thumb and fingers of the prosthetic hand. When the palm-side wire is pulled and the back-side wire is allowed to relax, the hand performs flexion. With the opposite wire operation, it performs extension.

**Figure 3:**
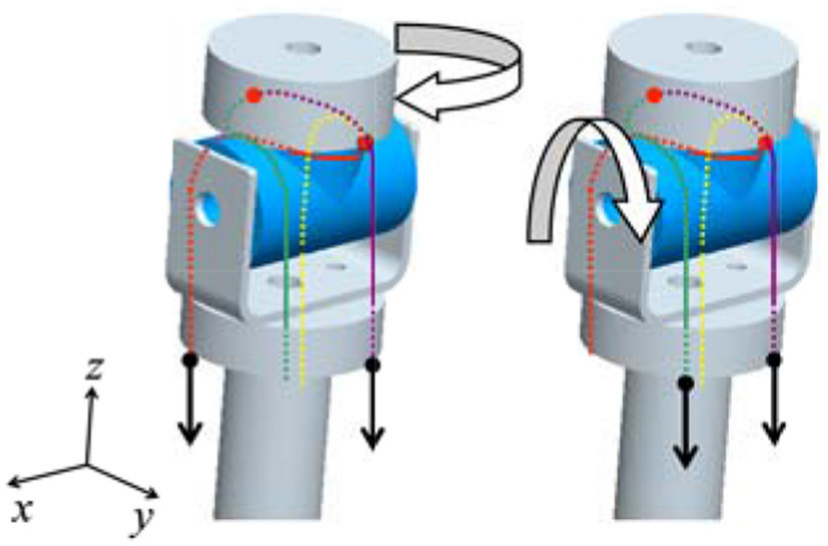
**Schematic diagram of the interference-based parallel-wire-driven mechanism**.

The joint mechanism, which has 2° of freedom in mutually orthogonal directions, is required for the wrist and upper arm joints. We thus invented an interference-based parallel-wire-driven mechanism that is hereafter referred to as a parallel wire mechanism. Figure 3 is a schematic diagram of the structure. It has two rotation mechanisms for *x*-axis and *z*-axis rotations. The cylindrical wire-guide leads the wires such that they are parallel to each other. Then, a rotating torque is generated around the *x*-axis by the synchronous traction of wires, and a rotating torque is generated around the *z*-axis by the asynchronous traction of wires in the same manner. To connect the wire symmetrically to the pulleys of the two motors, the interference power of the two motors is assigned for each degree of freedom.

**Figure 4:**
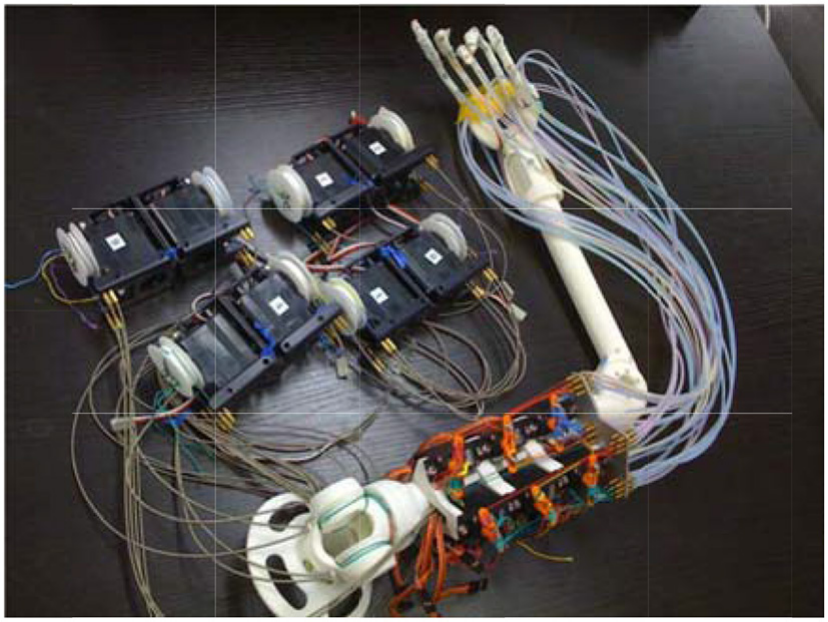
**The prosthetic arm with an interference-based wire-driven mechanism**.

These two types of interference-based wire-driven mechanisms were applied to develop a prosthetic hand and arm as shown in Figure 4. The shoulder joint of this arm has 2° of freedom in motion, flexion/extension and adduction/abduction, and the elbow joint has 2° of freedom, flexion/extension and internal rotation/external rotation.

It is important to consider the latency caused by power transmission through the wire when controlling prosthetic arms with a wire-driven mechanism. Because the wire is not rigid, power transmission latency is inevitable. As mentioned above, the latency of the prosthetic arm adopted in this study was about 200 ms. Here, the control operation delay was eliminated due to the brain activities preceding the appearance of motion.

### Modeling of the reaching task

**Figure 5:**
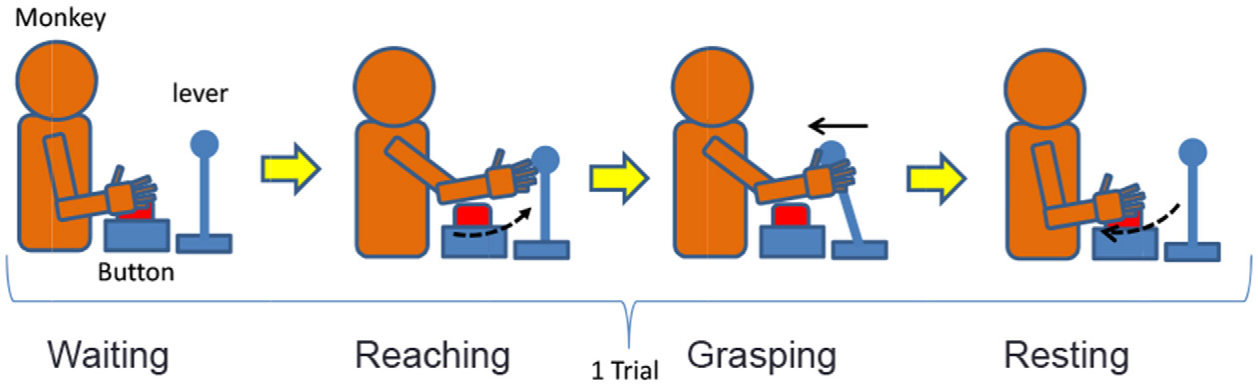
**A model of the reaching task**.

We designed a lever operation task as a reaching task based on the self-feeding motion of monkeys. Figure 5 shows an outline of the task, and Table 2 describes the monkey's different movement states during the task. The monkey was kept under restraint in a chair. First, a push button (home button) was set up under the monkey's right hand, and a lever was placed in front of the monkey. A tube was introduced into the mouth of the monkey, and liquid reward was given through a pump. The pump was triggered when the monkey pulled the lever after the home button was pushed. The monkey was adequately trained in performing this task.

**Table 2:** **Description of the monkey's states**.

**Symbol**	**State**	**Description**
ω_1_	Waiting	The monkey is pushing the home button
ω_2_	Reaching	The monkey is reaching its hand to the lever
ω_3_	Grasping	The monkey is grasping the lever and pulling it
ω_4_	Resting	The monkey is resting its arm and returning its hand on the home button

### Preprocessing of the EMG signals

The measured EMG signals were transformed into integrated EMGs (iEMGs) as follows:

(1) $$M_{m}(t)=\frac{1}{T_{\text{W}}}\sum_{\tau =0}^{T_{\text{W}}-1}|S_{m}(t-\tau )|,$$

where *S_m_*(*t*) is the signal measured by the *m*-th EMG electrode at time step *t*, *M_m_*(*t*) is the iEMG of the *m*-th channel of the EMG, and *T_W_* is the term of consideration. Because iEMGs strongly correlate with the exerted muscular force and are robust to white noise, they serve as appropriate indices of muscle activity.

### Preprocessing of ECoG signals

Some frequency bands are effective for determining the locomotive state of a subject (Sato et al., 2012). Table 3 shows the range of each frequency band.

**Table 3:** **Range of each frequency band**.

**Band**	**Range [Hz]**
Alpha α	7–11
Beta β	20–30
High-gamma 1 γ_L_	80–150
High-gamma 2 γ_H_	150–250

Previous studies have shown that high-gamma power strongly correlates to locomotive events in the same way as electroencephalography or local field potentials (LFPs). However, the range of the high-gamma band used differed in previous studies. For example, 60–200 Hz was used in a study assessing macaque LFPs and their potential implications in ECoG (Ray et al., 2008). On the other hand, another study used the frequency band of 80–150 Hz (Yanagisawa et al., 2011). To cover these different definitions, we separated the high-gamma band (80–250 Hz) into 2 ranges: γ_L_ (80–150 Hz) and γ_H_ (150–250 Hz). However, in our experimental setting (Western Japan), hum noise superimposed on the frequency band of 60 Hz. Therefore, the frequency band was trimmed at around 60 Hz. Additionally, the upper limit was decided according to the Nyquist frequency of our data acquisition system. The power of each band was determined by calculating the power spectrum with a short-time Fourier transform. The window size *L* equaled 128.

### Estimation of EMGs from ECoGs by a partial least squares regression

We estimated the EMGs from ECoG signals by a partial least squares (PLS) regression (Wold, 1975). Because of the relationship between the spatial resolution of the ECoG electrodes and the distances between the adjacent electrodes, the signals obtained from the electrodes were collinear. In the regression analysis, the collinearity made it difficult to determine the values of the regression coefficients and reduced the prediction accuracy. However, the PLS regression served to remove the collinearity and improved the precision of the regression analysis.

In this study, a PLS regression was performed with the following procedure. First, the feature vectors of the ECoGs were constructed as follows:

(2) $$x_{i}(t)=\left(\begin{array}{l} \alpha (i,t)\\ \beta (i,t)\\ \gamma_{\text{L}}(i,t)\\ \gamma_{\text{H}}(i,t) \end{array}\right),$$

(3) $$x(t)=\left(\begin{array}{c} x_{1}(t)\\ x_{2}(t)\\ \vdots \\ x_{N}(t) \end{array}\right),$$

where *x_i_*(*t*) (*i* = 1, 2, …, *N*) is the subvector of the feature vector *x*(*t*). Moreover, α(*i,t*), β(*i,t*), γ_L_(*i,t*), and γ_H_(*i,t*) are frequency band's power defined in Table 3 of channel *i* at time *t*. Namely, each element of *x_i_*(*t*) indicates the power of the corresponding frequency band. These elements are considered explanatory variables in the PLS regression. The regression model is as follows:

(4) $$y(t)=\beta_{0}+\sum_{k=1}^{r}\beta_{k}{x^{\prime }}_{k}(t)+E(t),$$

(5) $$x^{\prime }(t)=Ax(t),$$

where *y*(*t*) is the iEMG of the target muscle at time step *t*, *x*′_*k*_(*t*) is the *k*-th element of the latent variable vector *x*′(*t*) corresponding to *x*(*t*), β_*k*_ (*k* = 0, …, *r*) is the *k*-th regression coefficient, and *E*(*t*) is the error term. By using the PLS regression, the coefficient matrix *A* to maximize the covariance of *y* and *x*′ is decided, and the vector *x*′(*t*) is calculated as Equation (5). Namely, the latent variables which express the relationship between *y* and *x_i_* are achieved as the vector *x*′.

### Pattern classification with a linear discriminant analysis

The linear discriminant analysis (LDA), developed by Fisher (1936), was applied to classify the ECoGs into the four motions defined in Table 2. The scatter matrix *S_c_* (*c* = 1, 2, 3, 4) was defined as follows:

(6) $$S_{c}=\sum_{x\in {\text{X}}_{\text{c}}}\left(x-{\bar{x}}_{c}\right){\left(x-{\bar{x}}_{c}\right)}^{\text{T}},$$

where *X_c_* is a dataset of *x*(*t*) in the class ω_*c*_, *x*_*c*_ is the average vector of data set *X_c_*, and *N_c_* is the size of *X_c_*. The within-class scatter matrix *W_ij_* is defined by Equation (7) with the 2 classes of ω_*i*_ and ω_*j*_, and the between-class covariance matrix *B_ij_* is defined with Equation (8).

(7) $$W_{ij}=S_{i}+S_{j}=\sum_{n=i,j}\sum_{x\in {\text{X}}_{c}}\left(x-{\bar{x}}_{n}\right){\left(x-{\bar{x}}_{n}\right)}^{\text{T}},$$

(8) $$B_{ij}=\sum_{n=i,j}^{N_{n}}\left({\bar{x}}_{n}-\bar{x}\right){\left({\bar{x}}_{n}-\bar{x}\right)}^{\text{T}},$$

Then, the evaluation function *J*(*w_ij_*), which indicates the separation performance, is defined by Equation (9).

(9) $$J(w_{ij})=\frac{w_{ij}^{\text{T}}B_{ij}w_{ij}}{w_{ij}^{\text{T}}W_{ij}w_{ij}}.$$

The LDA yields the transform coefficient vector *w_ij_* by maximizing the evaluation function *J*(*w_ij_*). The discrimination function is defined as *g_ij_*(*x*(*t*)) in order to discriminate class ω_*i*_ from ω_*j*_ by using the transform coefficient *w_ij_* as follows:

(10) $$\{\begin{array}{l} x(t)\in \omega_{i}\Rightarrow g_{ij}(x(t))>0\\ x(t)\in \omega_{j}\Rightarrow g_{ij}(x(t))<0 \end{array}\;,$$

(11) $$g_{ij}(x(t))=w_{ij}^{\text{T}}x(t)+w_{ij}.$$

For multiclass classification, *ĉ*(*t*) the class at time step *t* is determined with Equation (12).

(12) $$\hat{c}(t)=\underset{i=1\ldots 4}{\text{argmax}}\sum_{j\neq i}^{g_{ij}}(x(t))$$

### Movement decision with the accumulated discrimination results

**Figure 6:**
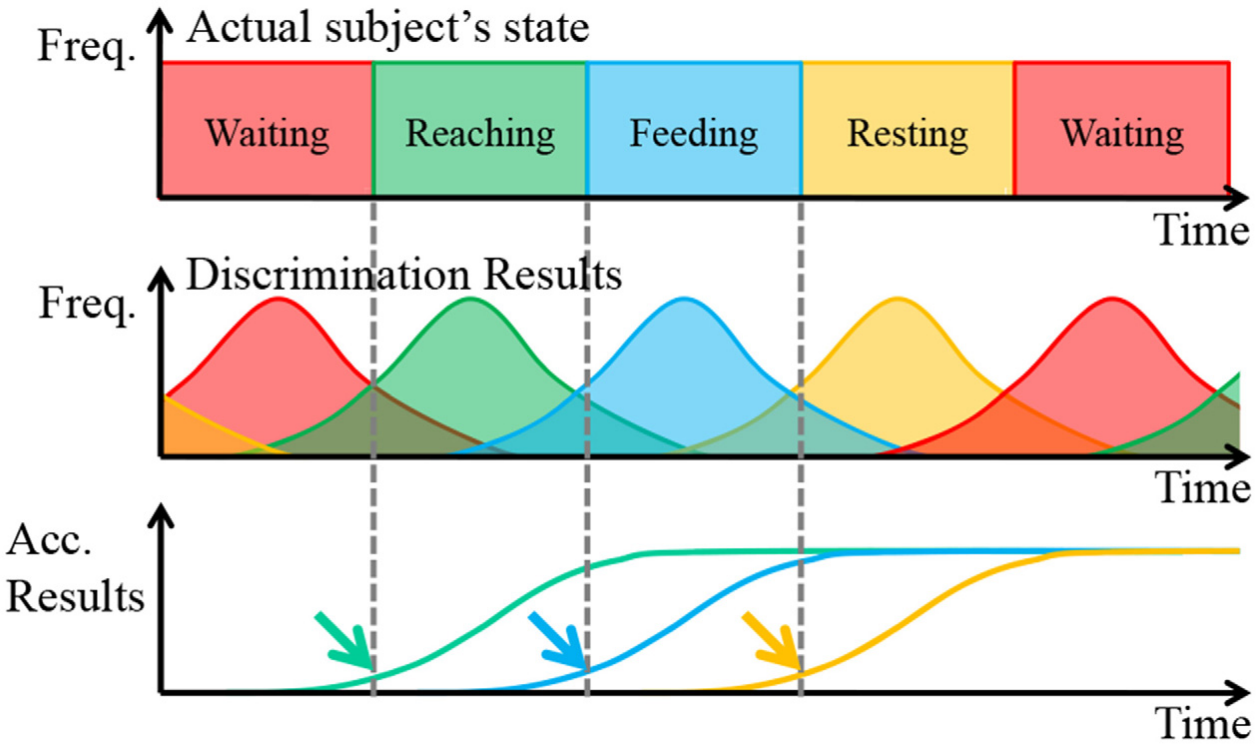
**Schematic diagram of the movement decision algorithm**. The solid line represents the actual values, and the dashed line represents the predicted values.

The movement of the prosthetic arm was determined with the results of the ECoG pattern discrimination. The discrimination results usually include misdiscrimination. Therefore, if the discrimination results directly reflect the control of a prosthetic arm, it can overdrive the arm. To avoid this problem, we performed movement decisions with the accumulated discrimination results. A schematic diagram of the algorithm is shown in Figure 6.

In Figure 6, Freq. is the abbreviation of frequency, and Acc. Results is the abbreviation of Accumulated Results, which is defined as the accumulated total of frequency of discrimination results. Focusing attention on the first row, the actual subject's states changed deterministically. In short, the frequency each state is always 100%. However, the discrimination results were probabilistic when considering misdiscrimination, and these often occurred around the point of state transition because the reaching task is a continuous motion. Finally, the accumulated discrimination results increased monotonically, and the upward trend began at the start of the subject's state transition.

With a proper threshold, the point of state transition, indicated by arrows, was estimated. The thresholds were determined by considering the difference in the start time and the speed of the prosthetic arm. In addition, the waiting state was treated another way. When the state of the prosthetic was determined to be waiting, the accumulated discrimination results of the other states expected that waiting would be reset. In addition, the accumulated discrimination results of waiting reset the state when it was determined not to be waiting. The transition of the prosthetic arm should be proper. Otherwise, it was assumed that the subject was performing irregular motions, such as grasping the bar of the cage, and so on. In such a case, the prosthetic arm stopped until the state changed to waiting.

### Trigger event generation according to the estimated EMGs

With the algorithm mentioned in the preceding section, stable control of the prosthetic arm was achieved with a latency of about 200 ms. The completion time was delayed even though the start time for movement of the prosthetic arm was almost the same as that for the monkey's actual movement. The performance of the monkey's own arm was superior to the prosthetic arm. In fact, the changes in the EMGs appeared before the changes in the motion, and, thus, preceding control became possible to determine the motion according to the estimated EMGs. Usually, it was difficult to reconstruct motion from EMGs. However, it was simple to generate a trigger from the EMGs under the presupposition that the state of the subject was waiting. The threshold processing of the EMGs of a certain muscle generated the trigger, and it was specified according to anatomical knowledge. As change of muscle activities should precede appearance of movement, it is possible to calculate a threshold that generates a trigger preceding appearance of movement. In this study, we used a threshold that canceled the prosthetic arm stable control latency mentioned above (200 ms).

### Ethical approval

All experimental procedures were performed in accordance with the Guidelines for Proper Conduct of Animal Experiments of the Science Council of Japan and approved by the Committee for Animal Experiment at the National Institutes of Natural Sciences (Approved No.: 11A157). The data presented for all experimental sessions were obtained from a female Japanese monkey (*M. fuscata*; body weight = 5.4 kg).

## Results

To confirm the usefulness of our proposed methods, we performed a number of experiments. First, the results of the EMG prediction with a PLS regression were determined to compare the predicted values to the actual values. Next, in order to compare the regular EMG pattern with the irregular one, we confirmed the stability of the predicted EMGs from the ECoGs. Finally, the results of the motion decision by using trigger event generation according to the predicted EMGs are shown.

### Comparison between the actual values and the predicted values of EMGs

In this experiment, we acquired a data sequence that included 100 regular trials. When the state transition of the monkey occurred in the sequence shown in Figure 6 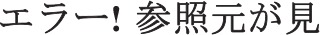

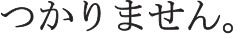
, the series of movements was counted as one trial. The coefficient matrix *A* was determined with data that included 90 trials, and the prediction accuracy was evaluated with sequential data that included 10 trials except for the data that were used to determine the coefficient matrix *A*. The 100 regular trials were divided into 10 groups of 10 trials each.

**Figure 7:**
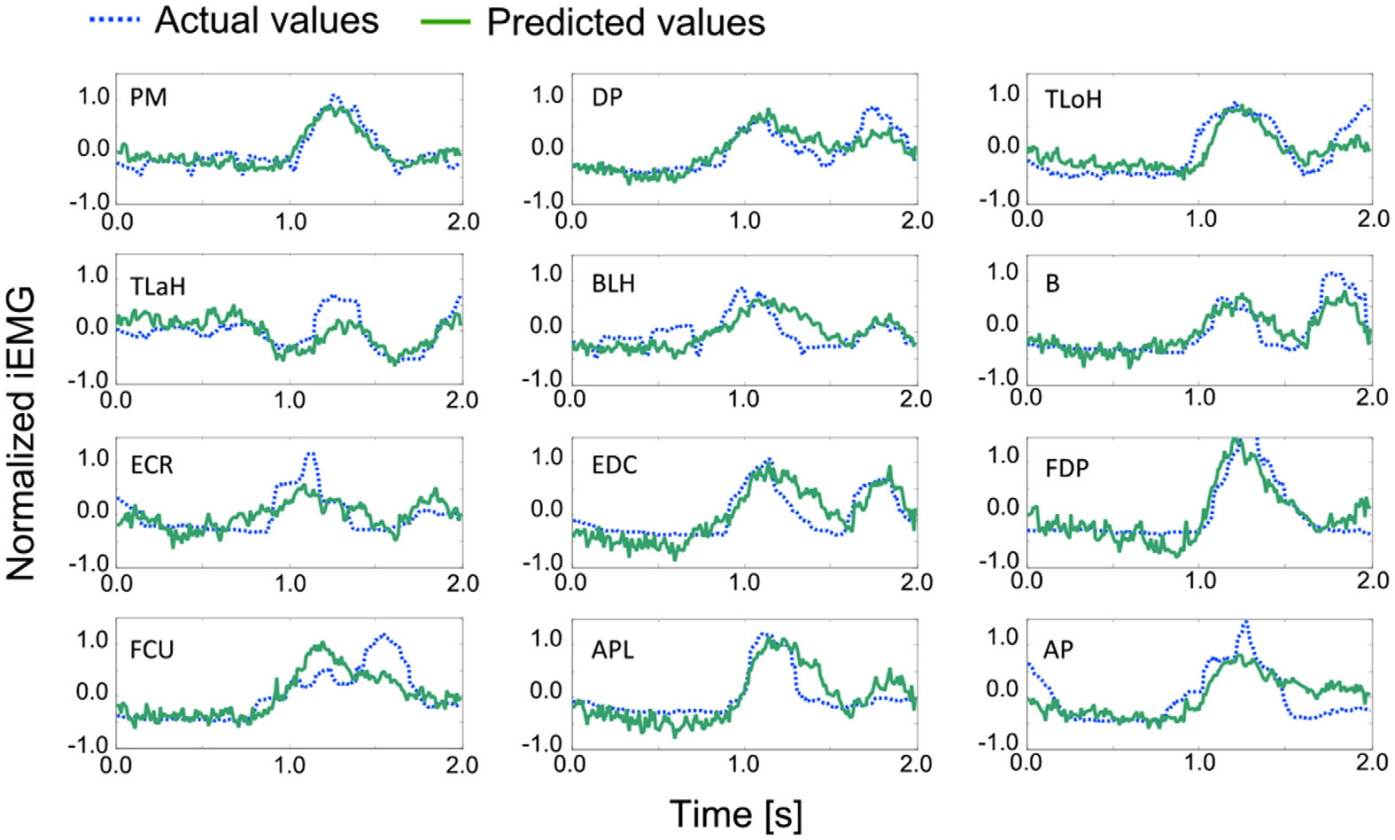
**Examples of the actual and predicted values for each muscle**. Table 1 contains the leg-end for all abbreviations. The dashed line represents the actual values, and the solid line represents the predicted values.

An example of a prediction over 2 s is shown in Figure 7. The solid line represents the actual values, and the dashed line represents the predicted values. In most cases, the trends and peak values were well matched. Although a peak time shift was seen in some cases, such as for the Flexor Carpi Ulnaris (FCU), rise times mostly matched.

For the quantitative evaluation, correlation factors and root-mean-square errors for each muscle are shown in Table 4. Correlation factors were calculated between actual values and predicted values. Student's *t*-test was performed under the null hypothesis that the correlation factor equals 0. Following this, it was confirmed that all correlation factors were significant at the 95% confidence level. Nine factors exceeded the correlation value and were considered highly correlated (0.7). In the case of the Triceps Lateral Head (TLaH), Biceps Long Head (BLH), and Extensor Carpi Radialis (ECR), the correlation values were not very high. However, they resulted in little difference compared to the root-mean-square error. We calculated them by applying leave-one-out cross-validation in one group selected from the 10 groups.

**Table 4:** **Comparative tables of correlation factors and root-mean-square errors for each muscle**.

**Correlation factors**			**Root-mean-square errors**		
PM	DP	TLoH	PM	DP	TLoH
0.88	0.83	0.82	0.12	0.17	0.20
TLaH	BLH	B	TLaH	BLH	B
0.59	0.55	0.85	0.21	0.21	0.16
ECR	EDC	FDP	ECR	EDC	FDP
0.64	0.79	0.89	0.18	0.21	0.13
FCU	APL	AP	FCU	APL	AP
0.75	0.83	0.71	0.20	0.19	0.18

Table 1 contains the legend for all abbreviations.

### Examples of irregular EMG patterns

**Figure 8:**
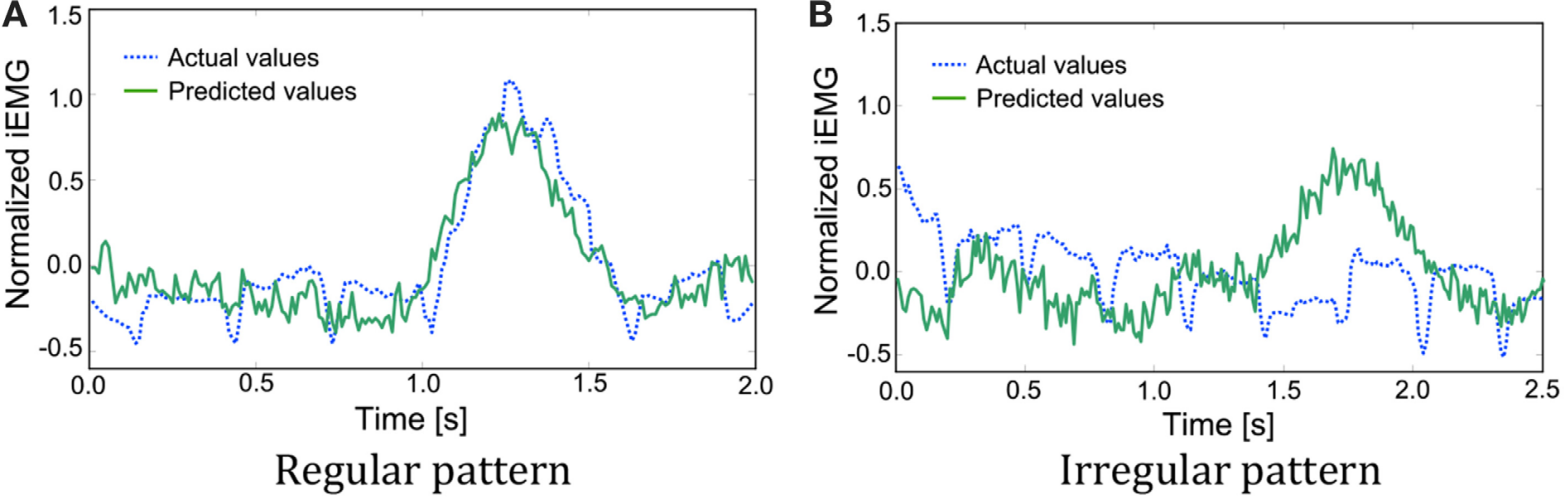
**Comparison of the actual and predicted integrated electromyography (iEMG) values of the pectoralis major with typical patterns**. **(A)** A regular pattern of the actual iEMGs and the predicted values. **(B)** An irregular pattern of the actual iEMGs and the predicted values.

As mentioned above, the iEMG prediction by PLS regression seemed to work well. However, some irregular patterns were found during the sequence that had period stability. The iEMG of the pectoralis major provides an illustrative example. Figure 8 shows the typical regular iEMG pattern and the irregular iEMG pattern during the 10 trials of continuous reaching motion. In 9 of the 10 trials, a regular pattern of the actual iEMGs was observed. However, in 1 trial, an irregular pattern was observed, as shown in Figure 8. Nevertheless, the reaching task was performed correctly in both of these cases. Specifically, it had period stability from a task-oriented point of view. Similarly, the brain activity also had stability. The waveforms of the predicted values were more similar to the regular actual iEMG patterns than to the irregular ones. Although the activity of the motor cortex was regular, the activity of the muscles that differed from the typical pattern was produced because of kinematic redundancy, as known as the degrees of freedom problem formulated by Bernstein (1967, 1996).

### Comparison among movement decision methods for prosthetic arms

**Figure 9:**
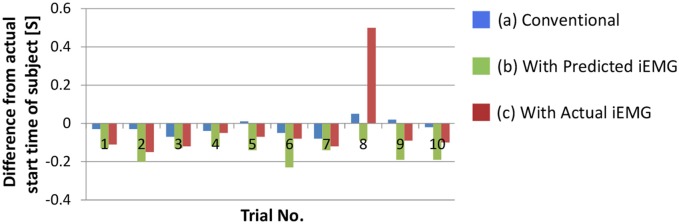
**Difference between the actual start time of the subject and the determined start time of the prosthetic arm**. **(A)** The result of the movement decision method that used accumulated discrimination results. **(B)** The result of the trigger generation with the predicted iEMG. **(C)** The results obtained with the actual iEMGs.

We confirmed the performance of the proposed movement decision method. To detect the start time of the upper arm movement, the deltoid posterior was selected because it increased monotonically with the upper arm movement. Figure 9 shows the difference between the actual start time of the subject and the start times that were determined with each method. In this section, the movement decision method that used accumulated discrimination results is treated as the conventional method (Figure 9A). Moreover, the result of the trigger generation with the predicted iEMG is shown as the proposed method (Figure 9B). The results obtained with the actual iEMGs are shown for comparison (Figure 9C).

**Figure 10:**
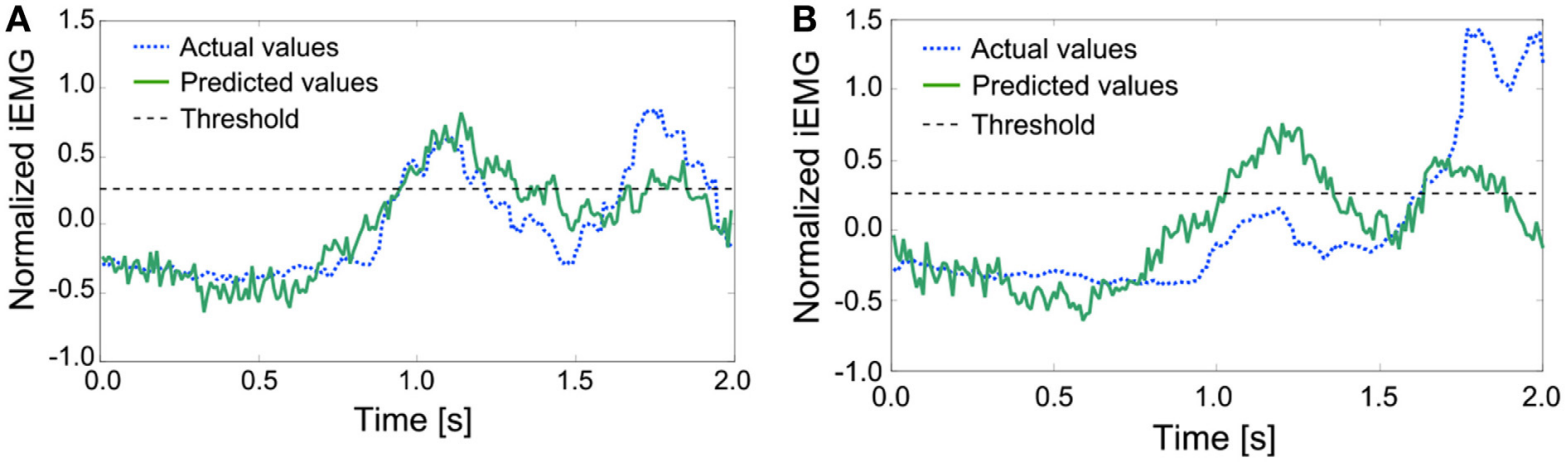
**Comparison of the actual and predicted iEMG values of the Deltoid Posterior**. **(A)** The actual and predicted values of the iEMG in the others. **(B)** The actual and predicted values of the iEMG in the 8th trial.

For the conventional method, differences in start times were almost 0. However, for the proposed method, each start time was earlier than that with the conventional method, that is, the response of the prosthetic arm was improved by the proposed method. In addition, the same method was adopted with the actual value of the iEMG instead of the predicted value. In almost all trials, the start time was earlier than that in the conventional method and was the same as the case in which the predicted value was used. However, a lengthy delay occurred in the 8th trial. To attempt to explain this phenomenon, the actual and predicted values of the iEMG in the 8th trial and the others are shown in Figure 10.

In the regular pattern, both the actual and predicted values had a diphasic trend, and the heights of the 2 peaks nearly aligned. However, in the 8th trial, the trends of the actual values and the predicted values differed from each other. Namely, the predicted values had trends that were the same as the regular pattern. The trigger event can be generated with a proper threshold (e.g., 0.25 as shown with dashed lines). However, the height of the first peak of the trend of the actual values was too low to generate a trigger event. In this case, a trigger event was generated at the second peak, resulting in a lengthy delay.

## Discussion

To construct a BMI prosthetic arm that performs a reaching task, it is preferable for the response to use information generated by muscle activity and not just the movement. Specifically, a trigger event is generated according to the predicted iEMGs from ECoGs by using a PLS regression. Additionally, motor intention can be correctly estimated by using the predicted value rather than the actual values for the control of a prosthetic arm. It is usually easier to estimate motor intention with muscle activities than with brain activities. At present, BMI prosthetic hands are not in practical use, while myoelectric prosthetic hands are already commercially available. This is because myoelectric prosthetic hands typically accomplish more sophisticated motions than BMI prosthetic hands do. However, in our current study, a converse phenomenon was observed. Our results indicate that during periodic movements, muscle activity predicted from brain activity is maintained using the periodicity rather than the actual muscle activity. To interpret this counterintuitive phenomenon, we describe the contribution of the cerebellum to motor function, which was clarified by Domen et al. (1998), as follows:

- When the environment is unstable and training for the locomotion is insufficient, feedback control is performed.
- When the environment is predictable and training is sufficient, feed forward control by the internal model constructed in the cerebellum is performed.

The brain modifies these two aforementioned modes correctly and achieves a task. Feedback control is executed to correct the error between target position and actual position. Because feedback delay can be several tens or hundreds of milliseconds, feedback control is applicable only to slow and primary motions. On the other hand, feed forward control is executed without feedback information from the sensory organs; it is performed according to the internal model constructed in the cerebellum. The monkey that was used as the experimental subject was well trained in the lever operation task. In other words, the monkey performed the motion that was “programmed” in its cerebellum. Then, the task-oriented motor intentions were decoded by the cerebral cortex. However, EMGs appeared due to information processing in the central nervous system, which was slower than in the cerebellum.

In conclusion, we found superior estimation of task-oriented motor intentions by constructing a BMI prosthetic arm. This was confirmed by comparing the periodicity of actual muscle activity with the estimated activity taken from brain ECoGs during the periodic movements of a monkey. Interestingly, if actual muscle activity became disordered, the estimated muscle activity maintained periodicity. Moreover, by comparing the time delay between the prosthetic arm control method based on actual muscle activity and the method based on estimated muscle activity, we found that the method using estimated muscle activity maintained greater stability than that using actual muscle activity.

### Conflict of interest statement

The authors declare that the research was conducted in the absence of any commercial or financial relationships that could be construed as a potential conflict of interest.
